# Timing the Generation of Distinct Retinal Cells by Homeobox Proteins

**DOI:** 10.1371/journal.pbio.0040272

**Published:** 2006-08-15

**Authors:** Sarah Decembrini, Massimiliano Andreazzoli, Robert Vignali, Giuseppina Barsacchi, Federico Cremisi

**Affiliations:** 1 Dipartimento di Biologia, Università degli Studi di Pisa, Pisa, Italy; 2 AMBISEN Center, High Technology Center for the Study of the Environmental Damage of the Endocrine and Nervous Systems, Universita' degli Studi di Pisa, Pisa, Italy; 3 Scuola Normale Superiore di Pisa, Pisa, Italy; Cambridge University, United Kingdom

## Abstract

The reason why different types of vertebrate nerve cells are generated in a particular sequence is still poorly understood. In the vertebrate retina, homeobox genes play a crucial role in establishing different cell identities. Here we provide evidence of a cellular clock that sequentially activates distinct homeobox genes in embryonic retinal cells, linking the identity of a retinal cell to its time of generation. By in situ expression analysis, we found that the three *Xenopus* homeobox genes *Xotx5b, Xvsx1,* and *Xotx2* are initially transcribed but not translated in early retinal progenitors. Their translation requires cell cycle progression and is sequentially activated in photoreceptors *(Xotx5b)* and bipolar cells *(Xvsx1* and *Xotx2).* Furthermore, by in vivo lipofection of “sensors” in which green fluorescent protein translation is under control of the 3′ untranslated region (UTR), we found that the 3′ UTRs of *Xotx5b, Xvsx1,* and *Xotx2* are sufficient to drive a spatiotemporal pattern of translation matching that of the corresponding proteins and consistent with the time of generation of photoreceptors *(Xotx5b)* and bipolar cells *(Xvsx1* and *Xotx2).* The block of cell cycle progression of single early retinal progenitors impairs their differentiation as photoreceptors and bipolar cells, but is rescued by the lipofection of *Xotx5b* and *Xvsx1* coding sequences, respectively. This is the first evidence to our knowledge that vertebrate homeobox proteins can work as effectors of a cellular clock to establish distinct cell identities.

## Abstract 2

A cellular clock mediated by sequential translational control of homeobox genes links retinal cell fates to their time of generation in *Xenopus.*

## Introduction

Different types of neurons are generated at predictable times in several developing brain structures [1–3]. Although the molecular machinery that links a type of nerve cell to its time of generation has been investigated in the fruit fly [4–6], little is known in higher animals. In the vertebrate retina, pluripotent progenitor cells generate the six main types of retinal neurons (ganglion, horizontal, cone, amacrine, rod, and bipolar cells) following an evolutionarily conserved time schedule [1]. This observation suggests that a molecular machinery has been selected to ensure tight coordination between cell birth date (that is the time of exit from the cell cycle) and the specification of a given neuronal cell fate [7].

Changes of retinal cell-fate competence (that is, the capability to generate one type of retinal cell rather than another) are controlled in time and space by the activity of proneural bHLH transcription factors [8]. Moreover, these alone are not sufficient to specify distinct cell fates [9], and several pieces of evidence suggest that they act in concert with homeobox gene products, which seem to refine their action to generate different cell types [10]. A number of homeobox genes were found to be necessary and/or sufficient to establish retinal cell identity: *prox1* is both necessary and sufficient for the generation of horizontal cells [11]; *Xbh1* promotes ganglion cells [12]; the *otx*-like *crx* [13] and *otx5* [14,15] support the generation and/or maintenance of photoreceptors; *vsx1* [16], *chx10*/*vsx2* [17], and *otx2* [14] sustain the production of bipolar neurons. While these data demonstrate the crucial role of homeobox genes in retinal cell identity, they do not address the question of how the neurogenetic timing is controlled. A critical question is therefore when, where, and how retinal homeobox genes are activated during retinal neurogenesis.

We recently observed that long-lasting cell cycle progression (and consequently a late cell birthday) is sufficient to generate late retinal cell types such as rods and bipolar cells [18]. Accordingly, the inhibition of cell cycle progression greatly enhances the capability of the retinal bHLH gene *Xath5* to support the generation of ganglion cells, which are the earliest-generated retinal cells, at the expense of bipolar cells, which are the latest-generated neurons [19]. These observations suggest that the activation of homeobox genes that are crucial for late retinal cell types may be linked to cell cycle progression rather than to absolute time. Notably, a similar mechanism occurs in *Drosophila,* in which the sequential expression of the transcription factors that control different neuronal identities requires cytokinesis [4].

The aim of this work is to provide evidence that the sequential activation of retinal homeobox genes depends on a cellular clock that establishes the cell birth dates of distinct retinal cell types. Here we report that the three *Xenopus* homeobox genes *Xotx5b, Xvsx1,* and *Xotx2* are translationally regulated with a timing that parallels that of the generation of photoreceptors *(Xotxb5)* and bipolar cells *(Xvsx1* and *Xotx2)* and that their translation depends on cell cycle progression. Moreover, we show that the block of cell cycle progression severely affects the generation of photoreceptors and bipolar cells, whereas Xotx5b and Xvsx1 proteins can overcome this effect. Our results confirm the importance of a cellular clock in establishing distinct cell fates and draw attention to the translational control of homeobox genes as a mechanism to regulate the neurogenetic timing in the *Xenopus* retina.

## Results/Discussion

**Figure 1:**
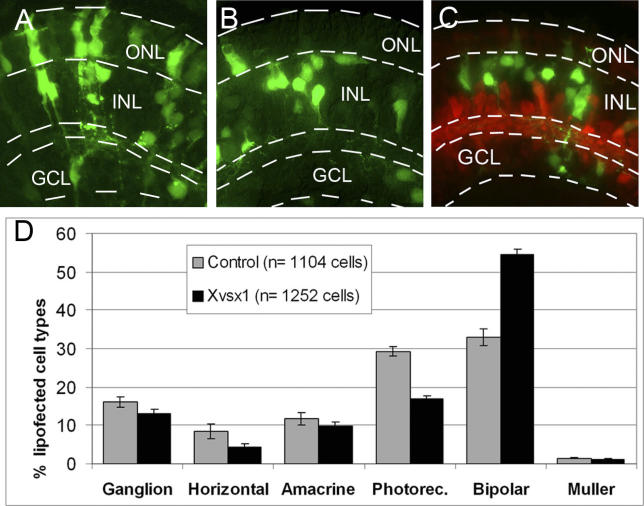
The *Xenopus Xvsx1* Homeobox Gene Supports Bipolar Cell Fate (A–C) Sections of st. 42-lipofected retinas. GFP (green) traces lipofection. (A) Example of control lipofection. (B and C) Example of *Xvsx1* lipofection. (C) Immunostaining (red fluorescence) with amacrine antibodies panel (anti-5-HT, anti-GABA, anti-tyrosine hydroxilase), labeling the main classes of amacrine cells at this developmental time [47,48] ONL: outer nuclear layer; INL: inner nuclear layer; GCL: ganglion cell layer. *Xvsx1* lipofection (B) increases the proportion of INL cells and decreases the proportion of ONL cells compared to control (A). The majority of the *Xvsx1*-lipofected cells in the INL are not stained either by amacrine markers (C), or by the horizontal marker *prox1* (not shown). (D) Statistical analysis showing the proportion of lipofected cell types. Cell types were identified as described [14]. Error bars indicate standard error of the mean. *Xvsx1* misexpression increases the proportion of bipolar cells (from 33% of control to 55%, student's *t*-test, *p* = 0.000043), mainly at the expense of photoreceptors (from 29% to 14%, student's *t*-test, *p* = 0.000011).

In the *Xenopus* retina, *Xotx2* is both necessary and sufficient for the generation of bipolar cells [14]. Moreover, while also *vsx1* [16] and *chx10*/*vsx2* [17] are necessary in other vertebrates, *chx10* is not sufficient to support the bipolar fate in *Xenopus* [20]. We recently isolated the *Xenopus* homolog of *vsx1, Xvsx1,* which is expressed by retinal progenitors and, in the mature retina, by bipolar cells (D'Autilia et al., unpublished data). To assay the ability of *Xvsx1* to support the generation of bipolar cells, we lipofected the *Xvsx1* coding sequence into stage (st.) 17–18 embryonic optic vesicles and compared the proportion of *Xvsx1*-lipofected cells to control-lipofected cells at the stage of mature embryonic retina (st. 42; Figure 1). As reported in Figure 1B and 1C, *Xvsx1* lipofection significantly increases the proportion of bipolar cells and decreases that of photoreceptors compared to control lipofection (Figure 1A and 1D). Thus, in the *Xenopus* retina, *Xotx5b* supports photoreceptor differentiation [14], while *Xotx2* and *Xvsx1* support bipolar cell differentiation.

**Figure 2:**
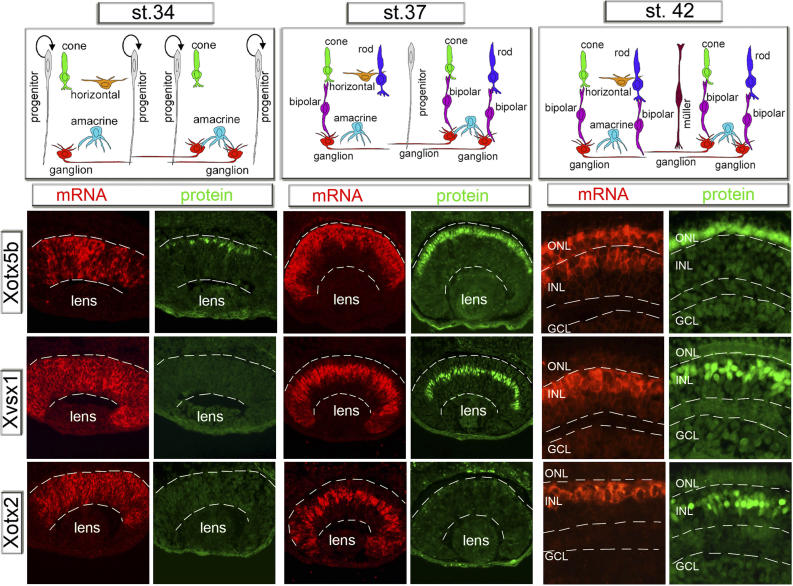
The Translation of the *Xenopus* Homeobox *Xotx5b, Xvsx1,* and *Xotx2* mRNAs Parallels the Generation of Photoreceptors and Bipolar Cells In situ hybridization of *Xotx2, Xotx5b,* and *Xvsx1* mRNAs (Fast Red detection) compared to immunostaining of the corresponding proteins (green detection) on serial 10-μm sections of embryonic retinas at st. 34 (mid-neurogenesis), st. 37 (late-neurogenesis), and st. 42 (mature embryonic retina). Schematics show the retinal cell types present at the corresponding times of analysis (see also Figure S2). Dashed lines border the entire thickness of neural retinas (st. 34–37), or indicate the boundaries between different cell layers (st. 42, magnification of central retinal aspect); GCL: ganglion cell layer, INL: inner nuclear layer, ONL: outer nuclear layer.

Photoreceptors (namely rods) and bipolar cells are the latest-generated retinal neurons both in *Xenopus* [9,21] and in mammals [1]. With the idea that *Xotx5b, Xotx2,* and *Xvsx1* were sequentially activated during retinal neurogenesis, matching the time of photoreceptor and bipolar cell generation, we examined their spatiotemporal pattern of expression. We observed that *Xotx5b, Xotx2,* and *Xvsx1* are strongly regulated at post-transcriptional level, both in time and space. At mid-retinal neurogenesis (st. 34 [22]), the mRNAs of these three genes show a similar widespread pattern of expression, but only the Xotx5b protein is detectable in few apical nuclei (Figure 2). Xvsx1 protein detection starts at late-retinal neurogenesis (st. 37), when the mRNAs of the three genes begin to segregate into specific retinal domains (Figure 2). Xotx2 protein is detectable at high levels from st. 38–39 (not shown) onward. The patterns of protein and mRNA expression are further refined in mature embryonic retinas (st. 42). At this time, *Xotx2* mRNA identifies most bipolar cells [14], *Xotx5b* mRNA marks photoreceptors and a sub-population of bipolar cells [14], and *Xvsx1* mRNA labels bipolar cells (D'Autilia et al., unpublished data)*.* At the protein level, Xotx2 and Xvsx1 are detectable in bipolar cells, whereas Xotx5b is visible in photoreceptors but not in bipolar cells. The peculiar post-transcriptional regulation of Xotx5b, Xvsx1, and Xotx2 is maintained in the ciliary marginal zone (CMZ), a proliferating region that, in the mature retina of fishes and amphibians, continues producing new retinal cells and recapitulates all the embryonic developmental steps [23]. Notably, CMZ analysis indicates that Xotx5b, Xvsx1, and Xotx2 proteins start to be detectable in early post-mitotic cells (Figure S1).

**Figure 3:**
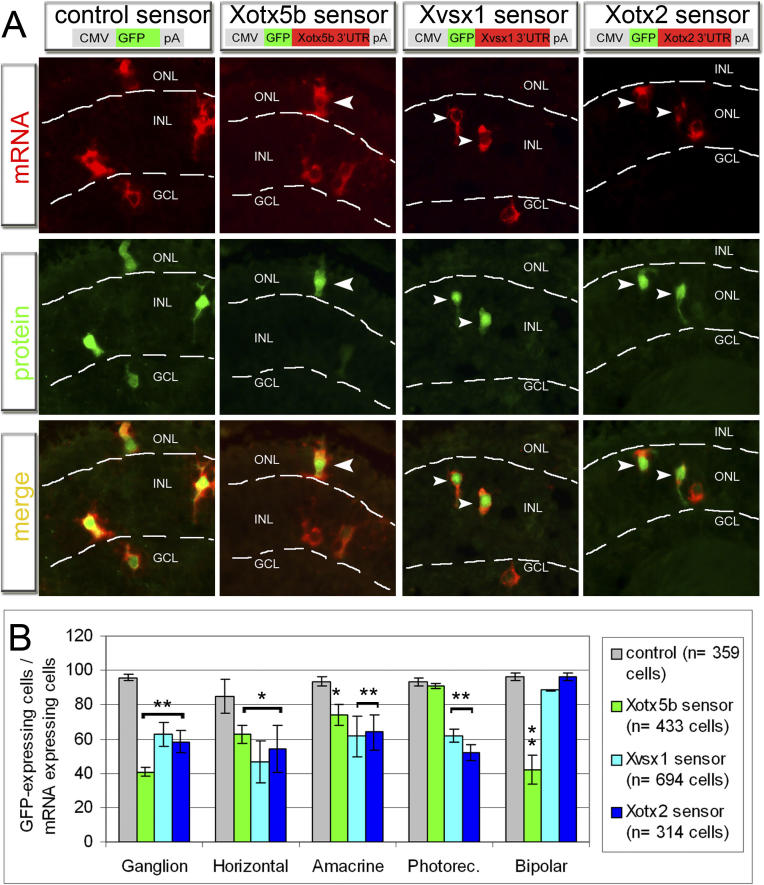
The 3′ UTRs of *Xotx5b, Xvsx1,* and *Xotx2* mRNA Direct Cell Type–Specific Inhibition of Translation (A) Detection of sensor mRNAs (Fast Red), sensor GFP protein (green immunodetection), or co-detection of both mRNA and protein (merge, yellow), in cells of mature retinas (st. 42) lipofected with GFP control vector, or UTR-carrying sensor vectors (see Materials and Methods). Unlike control GFP, sensor GFP translation is detectable (arrowheads) mainly in photoreceptors (*Xotx5b* sensor) or bipolar cells (*Xvsx1* and *Xotx2* sensors). ONL: outer nuclear layer; INL: inner nuclear layer; GCL: ganglion cell layer. (B) Bars show the proportion of sensor-translating/sensor-transcribing cell types. Number of cells is indicated by *n.* Error bars represent standard error of the mean. Single asterisk indicates *p* ≤ 0.05; double asterisk indicates *p* ≤ 0.01 (student's *t*-test).

What are the mechanisms controlling protein expression? We found that *cis*-acting signals in the 3′ UTR of *Xotx5b, Xvsx1,* and *Xotx2* mRNAs are sufficient to regulate their pattern of translation. We constructed sensors [24] in which the 3′ UTR of each gene was placed downstream of green fluorescent protein (GFP) cDNA (see Materials and Methods). We lipofected these sensors into the optic vesicles and analyzed the distribution of both the GFP mRNA and protein produced by the sensor in single lipofected cells. Figure 3A shows typical results of analyses performed on sections of st. 42 retinas. Control-lipofected cells show co-expression of mRNA and protein. Conversely, sensors are generally poorly translated, except in photoreceptors (*Xotx5b* sensor) or bipolar cells (*Xvsx1* and *Xotx2* sensors).

The inhibition of GFP translation driven by these UTRs in specific cell types is statistically significant, as reported in Figure 3B. Recently, extensive bioinformatic analyses have shown that at least 20% of the vertebrate genes display in their 3′ UTR a family of highly conserved short regions [25], most of which are complementary to a newly discovered class of regulatory short RNAs called microRNAs (miRNAs). Accordingly, *cis*-acting signals controlling protein translation through the binding of a miRNA have been found in the 3′ untranslated region (UTR) of Hox genes [26]. Using an in silico approach, we found that the 3′ UTR of *Xotx5b, Xvsx1,* and *Xotx2* contains candidate miRNA domains for 42 distinct miRNAs, four of which are shared by all the three 3′ UTRs (Table S1). These miRNA domains are widely dispersed in the 3′ UTR of each gene. Whereas such miRNA domains could reveal a functional relevance, specific RNA-binding proteins might also be involved in regulating the translation of retinal homeobox genes, since several of them have been shown to control the development and plasticity of the central nervous system, including the retina, in both *Drosophila* and vertebrates [27–29]. Thus, we found that the 3′ UTRs of *Xotx5b, Xvsx1,* and *Xotx2* are *cis*-acting regulators of translational repression, but the molecular nature of the *trans*-acting repressor(s) remains to be established.

**Figure 4:**
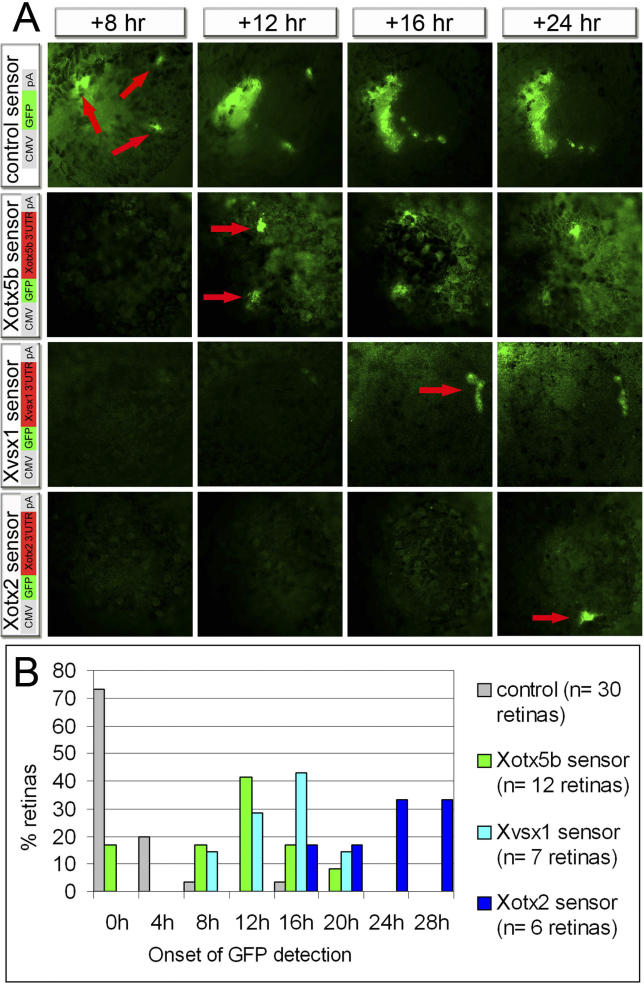
The 3′ UTRs of *Xotx5b, Xvsx1,* and *Xotx2* mRNAs Direct Time-Dependent Inhibition of Translation (A) Examples of time-lapse imaging of lipofected retinas. Times are calculated starting from st. 30 [22] (which corresponds to time 0). To better visualize GFP, pigmentation was abolished as described [43]. Each micro-photograph shows the entire area of a lipofected eye and is focused on lipofected cells of the neural retina. Red arrows point to lipofected clones of cells. (B) Statistical analysis of 68 records. Bars express the proportion of lipofected retinas in which GFP was first detectable at a given time. Number of retinas examined is indicated by *n.*

In addition to the role of the 3′ UTRs in patterning protein translation, we analyzed their effects on the timing of sensor translation by time-lapse imaging (Figure 4). At st. 30 (assumed as time 0 of imaging), almost all control-lipofected retinas already show GFP-positive clones. The onset of GFP detection in sensor-lipofected retinas is delayed and parallels the onset of detection of the corresponding proteins, peaking at 12 h (st. 35) for *Xotx5b* sensor, 16 h (st. 37) for *Xvsx1* sensor, and at 24 h (st. 39) for *Xotx2* sensor. These results show a correlation between the translational onset of a sensor and that of the corresponding gene. An obvious question is whether such timing associates with the cell birth dates of photoreceptors and bipolar cells. By BrdU labeling from st. 30, 34, and 37 and analyses of the BrdU-positive cells in mature retinas, we evaluated the proportion of dividing progenitors fated to generate a given cell type (Figure S2). The proportion of dividing photoreceptor progenitors drops from 68% (± 2.6% standard error of the mean) at st. 30 to 24% (± 3.3% standard error of the mean) at st. 34, when Xotx5b protein is first detectable. The percentage of proliferating bipolar progenitors falls from 63% (± 5.1% standard error of the mean) at st. 34 to 7% (± 1.2% standard error of the mean) at st. 37, when Xvsx1 protein is first detected, followed soon after by Xotx2 protein detection (st. 38–39). Thus, there is a temporal correlation between the translational onset of the three genes, the translational onset of sensors, and the cell birth dates of photoreceptor and bipolar cells.

**Figure 5:**
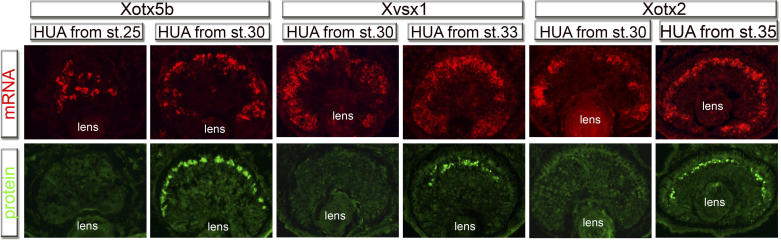
Blocking Cell Cycle by HUA Inhibits the Sequential Translation of *Xotx5b, Xvsx1,* and *Xotx2* mRNAs In situ hybridization (mRNA: Fast Red detection) and antibody immunodetection (protein: green fluorescence) on 10-μm thick serial sections of st. 42 retinas. Embryos were grown in medium containing HUA (hydroxyurea, 150 μM and aphidicolin, 20 μM) from the stage indicated to st. 42. The examples show typical results obtained in three different experiments (*n* > 15 embryos/treatment in each experiment).

We investigated the role of cell cycle progression in the translational control. We found that *Xotx5b, Xvsx1,* and *Xotx2* mRNAs require progressively increasing times of cell cycle progression to be efficiently translated*.* To establish this point, we blocked cell cycle progression by hydroxyurea/aphydicoline (HUA) [30]. HUA treatment (150 μM hydroxyurea, 20 μM aphydicoline) starting from st. 25, 30, 33, or 35 does not inhibit the transcription of *Xotx5b, Xvsx1,* and *Xotx2* genes (Figure 5). However, the corresponding proteins are detectable at the end of treatment (st. 42) only when this starts after st. 30 (Xotx5b), st. 33 (Xvsx1), or st. 35 (Xotx2). When detectable, the pattern of expression is comparable to that of control retinas (compare Figure 5 to st. 42 in Figure 2). Although impairing proper retinal lamination, HUA does not interfere with neural cell differentiation [30]. Accordingly, our analysis shows that retinas treated with HUA can express both general neuronal *(neurotubulin)* and glial (R5 antigen [31]) cell markers (Figure S3). Treatment from st. 30 does not affect expression of markers for ganglion cells (*hermes* [32]), horizontal cells (*prox1* [11]), amacrine cells (tyrosine hydroxilase+, GABA+, 5-HT+), and photoreceptors (interphotoreceptor retinoid-binding protein [IRBP] gene *[IRBP]* [33]). However, treatment from st. 25 dramatically decreases the expression of *IRBP,* as well as of amacrine markers (unpublished data).

**Figure 6:**
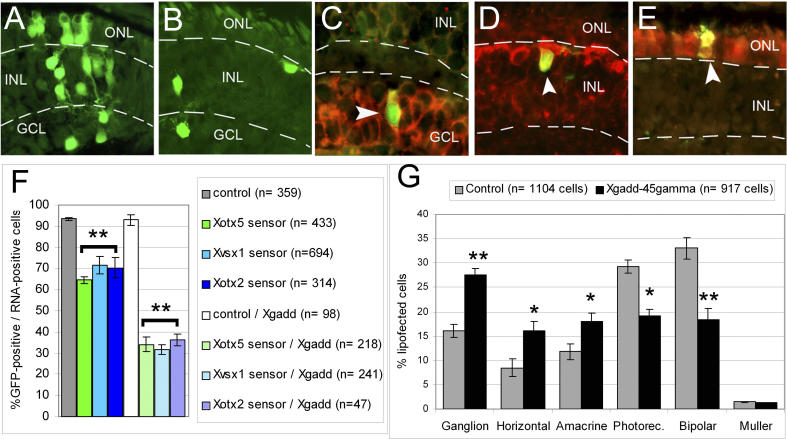
Cell Cycle Inhibiton by *Xgadd-45γ* Lipofection Affects Translation and Cell Fate (A–E) Retinal sections (st. 42) of control-lipofected retinas (A) or *Xgadd-45γ*–lipofected retinas (B–E). *Xgadd-45γ* lipofection decreases the size of lipofected cell clusters. (C–E) Show lipofected cells (GFP-traced) counterstained (arrowheads) with cell type markers: (C) ganglion cells (Fast Red mRNA detection of *hermes*), (D) horizontal cells (Fast Red mRNA detection of *prox1*), (E) cones (calbindin red immunodetection). ONL: outer nuclear layer; INL: inner nuclear layer; GCL: ganglion cell layer. (F and G) Bars indicate the proportions of lipofected cells translating sensors (F). Bars showing the proportions of lipofected cell types at st. 42 are indicated by (G). Number of counted cells is indicated by *n;* single asterisk indicates *p* ≤ 0.05; double asterisk indicates *p* ≤ 0.01 (student's *t*-test); error bars: standard error of the mean.

Is an early block of cell cycle progression sufficient to inhibit the translation of *Xotx5b, Xvsx1,* and *Xotx2* in a cell-autonomous way? To test this hypothesis, we inhibited cell cycle progression of single retinal progenitors in a normal environment by the lipofection of the cell cycle inhibitor *Xgadd-45γ* [34,35] (Figure 6). When overexpressed in medaka fish early blastula, *Xgadd-45γ* favors cell cycle exit in G1 [34]. In the *Xenopus* retina, it is directly induced by *Xath5* and expressed by retinal progenitors about to exit from the cell cycle [36]. Misexpression of *Xgadd-45γ* inhibits retinal cell divisions: BrdU injected at st. 33–34 is detected at st. 42 in fewer *Xgadd-45γ*–lipofected cells (19% ± 3.4% standard error of the mean) compared to control-lipofected cells (41% ± 0.79% standard error of the mean; *p* = 0.001, student's *t*-test). Significantly, *Xgadd-45γ*–lipofected cells translate *Xotx5b, Xvsx1,* and *Xotx2*–co-lipofected sensors less efficiently than cells lipofected with the sensor alone (Figure 6F). Moreover, the number of *Xgadd-45γ*–lipofected cells that express Xotx5b, Xvsx1, and Xotx2 proteins is considerably lower than that of control cells (not shown). In addition, *Xgadd-45γ* misexpression decreases the proportion of photoreceptor and bipolar lipofected cells compared to control (Figure 6A–E and 6G).

**Figure 7:**
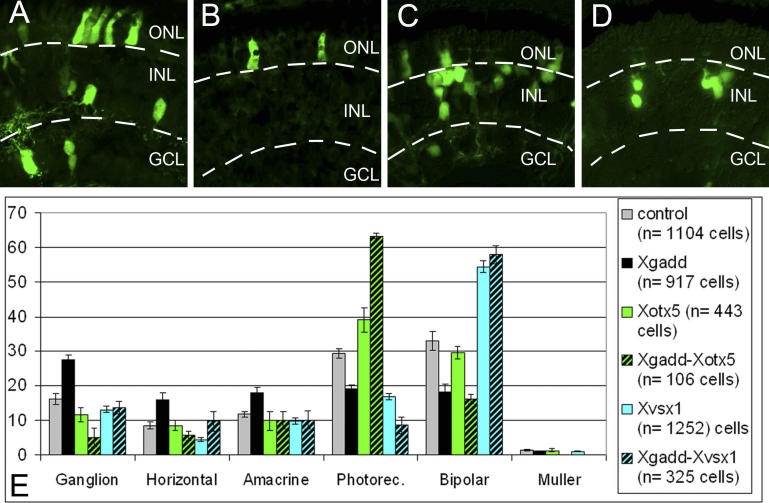
*Xotx5b* and *Xvsx1* Counteract the Inhibitory Effects of *Xgadd-45γ* on the Differentiation of Photoreceptors and Bipolar Cells (A–D) Examples of retinal sections from embryos lipofected with *Xotx5b* (A), *Xotx5b* and *Xgadd-45γ* (B), *Xvsx1* (C), and *Xvsx1* and *Xgadd-45γ* (D). ONL, outer nuclear layer; INL, inner nuclear layer; GCL, ganglion cell layer. (E) Statistical analysis showing the proportion of lipofected cell types after lipofection of the constructs indicated in legend. Number of lipofected cells is indicated by *n.* Error bars indicate standard error of the mean.

We propose that it is the decreased production of Xotx5b and Xvsx1 proteins after the block of cell cycle progression that causes the decrease of photoreceptor and bipolar cells. If such is the case, then the effect of *Xgadd-45γ* should be reversed by Xotx5b and Xvsx1 proteins. In fact, the co-lipofection of the *Xotx5b* coding region (without 3′ UTR) with *Xgadd-45γ* raises the fraction of photoreceptors from 19% (± 1.3% standard error of the mean) to 63% (± 0.9% standard error of the mean; *p* = 0.0000025, student's *t*-test) and that of *Xvsx1* increases the proportion of bipolar cells from 18% (± 2.2% standard error of the mean) to 58% (± 2.5% standard error of the mean; *p* = 0.00002, student's *t*-test) (Figure 7).

On the opposite, the co-lipofection of *Xotx5b* or *Xvsx1* constructs, including the corresponding 3′ UTRs, is drastically less effective in rescuing the proportion of photoreceptors cells (*Xotx5b* coding + 3′ UTR: 31% ± 2.1% standard error of the mean) and bipolar cells (*Xvsx1* coding + 3′ UTR: 29% ± 1.8% standard error of the mean) compared to the co-lipofection of the coding sequences alone (Figure S4). Indeed, the 3′ UTR included in these constructs is the same as that which is responsible for the significant decrease of translation when assayed by GFP sensors both in normal (Figures 3 and 4) and in *Xgadd-45γ*–arrested cells (Figure 6F). According to the antiproliferative effect elicited by *Xgadd-45γ,* the size of clusters of cells co-lipofected with *Xgadd-45γ* and *Xotx5b* (Figure 7B), or with *Xgadd-45γ* and *Xvsx1* (Figure 7D), was always smaller than the size of clusters of cells lipofected with only *Xotx5b* (Figure 7A), or with *Xvsx1* (Figure 7C)*.* Remarkably, the majority of bipolar cells generated by the co-lipofection of *Xgadd-45γ* and *Xvsx1* have an earlier cell birth date than control-lipofected bipolar cells, as indicated by BrdU-incorporation experiments (not shown). These results indicate that the overexpression of Xotx5b and Xvsx1 proteins can bypass a cell cycle–dependent cellular clock that sets the generation of photoreceptors and bipolar cells. However, these proteins are unlikely elements of the cell clock machinery, because they are not expressed at a detectable level in cycling cells (Figure S1). Rather, our data suggest that they are downstream effectors of this clock, which could set the time of neuronal generation by translational inhibition.

How would this cellular clock measure the time to set the generation of late cell types? Previous studies in cortical development [37] and in rat retinal development [38] have shown that the cell cycle length of neural progenitor cells increases over time. Because these progenitors generate different cell types over time, this suggests a correlation between cell cycle length and cell fate. By a labeling-index (LI) analysis [39], we estimated that the cell cycle length of mid-neurogenesis retinal progenitor cells (st. 30) is 5.1 h (± 1.3 h), whereas that of later progenitors (st. 34) is 8.1 h (± 0.6 h; Figure S5A).

**Figure 8:**
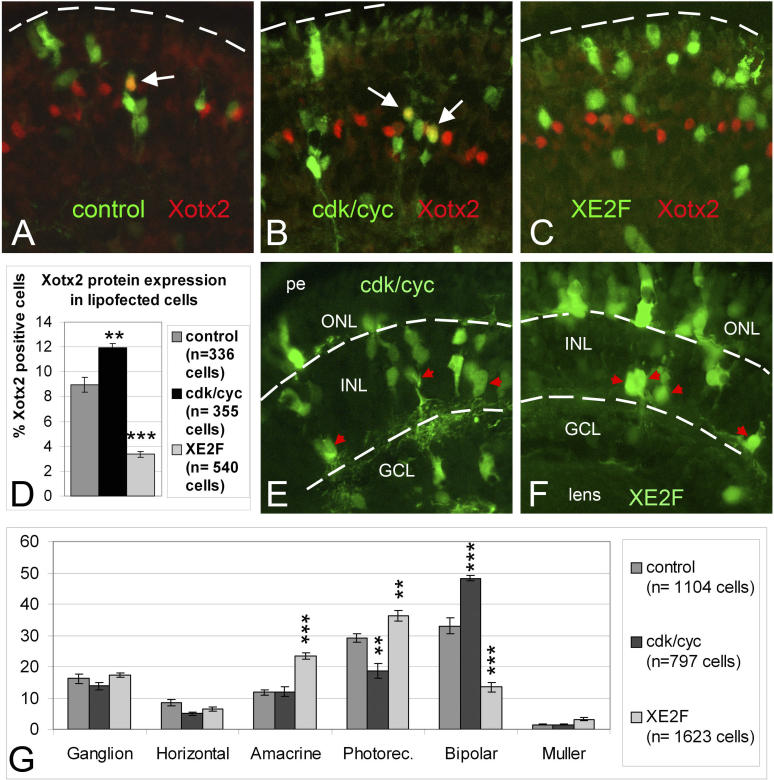
*XE2F* Lipofection Inhibits Xotx2 Translation and the Generation of Bipolar Cells (A–C) st. 40-lipofected retinas. Lipofected cells are traced by GFP (green), Xotx2 immunostaining in red. Arrows indicate Xotx2-positive lipofected cells. (D) Histogram showing the proportion of retinal lipofected cells expressing Xotx2 protein. Number of counted cells is indicated by *n.* Double asterisk indicates *p* = 0.009; triple asterisk indicates *p* = 0.0001 (student's *t*-test); error bars indicate standard error of the mean. (E–G) Cell type analysis of lipofected retinas at st. 42. Examples are shown of retinas lipofected with *cdk2/cyclinA2* (cdk/cyc in [E]) and *XE2F* (F). GCL: ganglion cell layer; INL: inner nuclear layer; ONL: outer nuclear layer; pe, pigmented epithelium. Red arrowheads point at cells with amacrine morphology, which represent the majority of cell types in the INL of *XE2F*-lipofected retinas. Bars in (G) show the proportion of lipofected cells types at st. 42. Number of counted cells is indicated by *n;* double asterisk indicates *p* ≤ 0.01; triple asterisk indicates *p* ≤ 0.001 (student's *t*-test); error bars: standard error of the mean. The lipofection of *cdk2/cyclinA2* and *XE2F* increases and decreases, respectively, the proportion of bipolar cells compared to control. The decrease of photoreceptors after *cdk2/cyclinA2* lipofection is due to a reduction of cones [18].

To assay a possible functional correlation between cell cycle length and cell fate, we shortened the cell cycle of late progenitor cells by lipofection of the cell cycle regulator *E2F,* which is necessary for cell cycle progression after mid-blastula transition [40]. At st. 34, cells lipofected with *XE2F* have an estimated cell cycle length of 5.5 h (± 1.2 h), whereas *cdk2/cyclinA2*–lipofected cells show a cell cycle length (7.8 ± 0.9 h) that is comparable to that of non-lipofected cells of the same age (Figure S5B). Nonetheless, more *XE2F*-lipofected cells (48% ± 2% standard error of the mean) and *cdk2/cyclinA2*–lipofected cells (45% ± 0.1% standard error of the mean) are cycling than control-lipofected cells (23% ± 0.1% standard error of the mean) at this stage (Figure S5B). Thus, both *XE2F* and *cdk2/cyclinA2* lipofection delay the exit from the cell cycle, but only *XE2F* lipofections shortens cell cycle length. Remarkably, Xotx2 translation at st. 40, soon after its normal onset, is considerably inhibited by *XE2F* lipofection compared to control, whereas it is slightly increased by *cdk2/cyclinA2* lipofection (Figure 8A–8D).

According to this observation, *XE2F*-lipofected cells generate significantly fewer bipolar cells than control-lipofected cells, whereas *cdk2/cyclinA2* lipofection increases their proportion (Figure 8E–8G). Since both *cdk2/cyclinA2* and *XE2F*-lipofected cells proliferate longer than control cells, their different cell fates cannot be explained in terms of absolute time spent as cycling cells. Rather**,** we speculate that a long cell cycle is necessary during the last cell division(s) of a progenitor to translate Xotx2 protein after cell cycle exit and to eventually differentiate as a bipolar cell. These results, together with the natural lengthening of cell cycle observed during retinogenesis, support the idea that the retinal cell clock would measure the cell cycle length, rather than time, to set the generation of late retinal cells.

### Conclusion

We showed that the *Xotx5b, Xvsx1,* and *Xotx2* mRNAs are sequentially translated during retinal neurogenesis. Translational control accounts both for the timing of activation and the cell-specificity of expression of the three genes. Our results show that cell cycle progression is necessary to sequentially remove the translational inhibition of *Xotx5b, Xvsx1,* and *Xotx2.* Consequently, homeobox proteins (Xotx5b, Xvsx1, or Xotx2) produced by a retinal post-mitotic cell depend on the cell birth date. Since these three homeobox proteins are crucial for establishing different cell types, this explains in molecular terms why cells that have different birth dates become different retinal neurons.

Our observations indicate that a cellular clock depending on cell cycle progression sets the generation of photoreceptors and bipolar cells and that Xotx5b, Xvsx1, and Xotx2 proteins are downstream effectors of such a clock. Although our observations suggest that this clock measures cell cycle length and that translational inhibitors are part of the clock machinery, its molecular nature is at present unknown. It is now crucial to extend these findings to other retinal cell-fate genes, to dissect the mechanisms of translational inhibition and to find out how cell cycle progression can remove the translational inhibition over developmental time.

## Materials and Methods

Embryonic developmental stages were evaluated as described by Nieuwkoop and Faber [22]. To immunodetect Xvsx1, Xotx5b, and Xvsx1 proteins, immunoaffinity-purified polyclonal antibodies were generated in rabbit by PRIMM SRL. Synthetic peptides (three for each of *Xvsx1* and *Xotx5b,* and two for *Xotx2*), corresponding to 15aa-long regions outside the homeobox of the predicted protein sequence, were used as immunogens. Antibody specificity was first assayed by Western blot analysis. Immunostaining of HEK 293T cells, transfected with the coding sequence of the three genes, confirmed the specificity of all three antibodies.

In situ hybridization and immunohistochemistry were performed according to Viczian et al. [14]. The following antibodies were used: anti-Xotx5b (1:20), anti-Xotx2 (1:200), anti-Xvsx1 (1:150), anti-calbindin (Merck Biosciences, http://www.merckbiosciences.co.uk/home.asp; 1:1,000), anti-5-HT (DiaSorin, http://www.diasorin.com; 1:1,000), anti-GABA (DiaSorin; 1:1,000), anti-tyrosine hydroxilase (DiaSorin; 1:1,000), R5 [31] (1:100, kindly supplied by W. Harris [University of Cambridge, Cambridge, United Kingdom]), anti-GFP (Molecular Probes, http://probes.invitrogen.com; 1:500), fluorophore-conjugated (oregon green, rhodamine) anti-mouse and anti-rabbit antibodies (Molecular Probes; 1:500). Antibodies were incubated as described [14] except anti-Xotx5b (0.3% instead of 0.1% Triton X-100) and anti-*Xvsx1* (antigen unmasking by 15-min treatment with 2N HCL).

GFP-sensors were obtained by cloning RT-PCR fragments of the 3′ UTRs into XhoI-XbaI sites of pCS2-GFP (http://sitemaker.umich.edu/dlturner.vectors), just downstream of the GFP coding sequence. Co-transcription of GFP and UTR sequences is under CMV promoter control (see schematics in Figure 2). The following fragments were inserted: *Xotx5b:* +1,097 + 2,193; *Xvsx1*: +1,482 + 2,878; *Xotx2:* +1,039 + 1,666; pCS2-GFP vector was used as negative control.

Lipofections [41] were carried out as described [14,18]. A Myc-tag-pCS2-*Xgadd-45γ* construct [35] suitable for lipofection was kindly provided by José Luis Gomez-Skarmeta (University of Murcia, Murcia, Spain). The pCS2-*XE2F* construct, containing the *Xenopus XE2F* coding region [42], was a generous gift of Ali Hemmati-Brivanlou (Rockefeller University, New York, United States). For sensor lipofections, sections were hybridized with a GFP Dig-labeled antisense probe; after Fast Red (Roche, Basel, Switzerland) detection of GFP mRNA, GFP protein was revealed by anti-GFP antibody.

In time-lapse experiments, lipofected retinas were imaged every 4 h, starting from st. 30 (considered as time 0). To avoid pigmentation, embryos were treated with 0.003% *N*-phenylthiourea (Sigma, St. Louis, Missouri, United States) [43] until imaging. After hatching and shortly before imaging (st. 28), embryos were embedded into soft agar (1%, in 0.1X MMR buffer) in 24-well plates, with their lipofected eyes up. Images of the lipofected eyes were obtained using epifluorescence stereomicroscopy (Nikon SMZ1500 [Tokyo, Japan]) connected with a digital camera (Photometrix CoolSnap, Roper Scientific, Trenton, New Jersey, United States). After imaging, some of the lipofected eyes were fixed, sectioned, and analyzed to confirm the nature of the transfected cells.

## Supporting Information

Figure S1 *Xotx5b, Xvsx1,* and *Xotx2* Expression in the CMZ Comparison between *Xotx5b, Xvsx1,* and *Xotx2* mRNA or protein detection (red) and BrdU-positive cells (green) in the CMZ of st. 42 retinas, after an 8-h BrdU pulse. Since this time of incorporation corresponds to the average cell cycle length of a late embryonic retinal progenitor (see Figure S5), the region of green-labeled cells reasonably excludes the majority of post-mitotic cells. From its most marginal aspect (M) towards the central side of the retina (C), the CMZ recapitulates the different stages of embryonic retinal neurogenesis [23], with more marginal cells earlier (less mature) than more central ones. Green arrows point at the central boundary of BrdU immunodetection, and red arrows indicate the marginal border of mRNA/protein detection. Whereas mRNA detection of the three genes always largely co-localizes with BrdU-labeled cells, none of the cells expressing detectable levels of the corresponding protein contains BrdU. These data indicate that *Xotx5b, Xvsx1,* and *Xotx2* start to be translated at a measurable level in post-mitotic cells.
(743 KB JPG)

Figure S2 Neurogenetic Timing in *Xenopus* Embryonic Retina To investigate the cell birth date (that is the time of exit from the cell cycle) of different retinal cells, we labeled dividing retinal progenitors by BrdU intrabdominal injections [19] from st. 30, st. 34, and st. 37, and analyzed their differentiation fates at st. 42 (mature embryonic retina). As the generation of the *Xenopus* Müller glia, which is the last retinal cell type to exit from the cell cycle, was extensively investigated both in terms of cell birth date and at the molecular level [31], we focused our attention on retinal neurons.
(A–C) Examples of st. 42 retinal sections immunostained for BrdU (green signal). Dashed lines enclose the central part of retina that was considered for statistical analysis.
(D–F) Magnifications of st. 42 retinal sections in which BrdU (green) was co-detected with specific retinal markers (Fast Red mRNA detection). White arrows point to double-labeled cells. The following markers were used to identify different cell types: *hermes* [32] for ganglion cells (D), *prox1* [11] for horizontal cells (not shown), amacrine antibodies panel (anti-5-HT, anti-GABA, anti-tyrosine hydroxilase) as in Figure 1 (not shown), *IRBP* [33] for the external segment of photoreceptors (E), *Xvsx1* (F), and *Xotx2* (not shown) for bipolar cells. ONL: outer nuclear layer; INL: inner nuclear layer; GCL: ganglion cell layer.
(G) Bars show the proportion of each cell type that was still dividing at the time of BrdU injection. Error bars show standard error of the mean. We classified the BrdU-positive cells according to their morphology, position in the retinal layers, and expression of markers. The cell birth dates of the different *Xenopus* retinal neurons are partially overlapping. Nonetheless, rods among photoreceptors [21] and bipolar cells show the latest cell birth dates. A substantial proportion of their progenitors are still dividing (being BrdU-positive) at st. 34 (24% photoreceptors and 63% bipolar). At this stage, only a few ganglion, horizontal, and amacrine progenitors are still dividing (6%, 5%, and 12%, respectively). Bipolar cells are the latest neurons, as the majority of them (63%) are still dividing at st. 34.
(820 KB JPG)

Figure S3 Effects of HUA Treatment on Retinal Histogenesis Retinal sections of st. 42 embryos, treated with HUA (150 μM hydroxiurea, 20 μM aphidicoline) from st. 30, compared to control. In situ hybridization of mRNAs *(neurotubulin, hermes, prox1,* and *IRBP)* are detected with Fast Red, and antibodies (anti-R5, amacrine antibodies panel) are immunodeteced with Oregon green–conjugated secondary antibody. According to Harris et al. [30], HUA blocks cell proliferation in 4 h from the beginning to the end of treatment, as detected by BrdU-incorporation assay and immunodetection of mitotic cells with the phosphorylated form of Histone3 (not shown). The treatment reduces retinal size but does not impede terminal cell differentiation, as shown by the expression of the Müller glial marker R5 [31] and *neurotubulin,* the staining of which in treated embryos is comparable to control. Immunostaining of the neuronal marker acetylated tubulin (Sigma T6793; 1:1,000) confirmed the observation obtained by in situ hybridization with a *neurotubulin* probe (not shown). The pattern of *neurotubulin* and Müller glial staining indicates that retinal layering is compromised. This happens, even more severely, when treating from earlier stages (not shown, compare to Harris et al. [30]). The expression of markers for ganglion cells *(hermes)* and horizontal cells *(prox1)* is not affected by treatment. The expression of markers for amacrine cells (amacrine antibodies panel as in Figure 1: anti-5-HT, anti-GABA, and anti-tyrosine hydroxilase) and photoreceptors *(IRBP)* is often reduced but is still detectable with a pattern similar to that of control in all the examined embryos. Treatment from st. 25 strongly reduces *IRBP* and amacrine markers, but allows the expression of *hermes* and *prox1* (not shown).
(609 KB JPG)

Figure S4 Functional Comparison between Constructs Carrying Coding and Coding Plus 3′ UTR of *Xotx5b* and *Xvsx1* in Co-Lipofection Experiments with *Xgadd-45γ*
Statistical analysis showing the proportion of lipofected cell types after lipofection of the constructs indicated in legend (Xgadd stays for *Xgadd-45γ*). “Full” indicates constructs containing the coding region plus its 3′ UTR. These constructs were assembled by cloning the complete 3′ UTR sequence (see Materials and Methods) upstream of the coding sequence in the pCS2 vector. Number of lipofected cells are indicated by *n.* Error bars indicate standard error of the mean. Asterisks show the statistical significance of the differences between coding and coding +3′ UTR–containing constructs: Single asterisk indicates *p* ≤ 0.05; triple asterisk indicates *p* ≤ 0.001.
(366 KB JPG)

Figure S5 LI Analysis (A and B) Analyses of the BrdU-LI (the proportion of BrdU-labeled cells) in wild-type (wt) (A) and lipofected (B) retinas. BrdU was injected and detected as described [19,31]. Images in (A) show examples of retinal sections of wt embryos, after different times of BrdU in incorporation (h BrdU) starting from st. 30 and st. 34. White lines demarcate the central retinal region that was considered for quantitative analysis. BrdU-positive nuclei are detectable in green among DAPI-stained (blue) nuclei. L: lens. Images in (B) show magnifications of retinas (bordered by dashed lines) lipofected at st. 17 with *cdk2/cyclinA2* (cdk/cyc [18]), or with *XE2F* [42], after BrdU cumulative incorporation from st. 34. White arrows point at BrdU-positive nuclei (detectable in red) of lipofected cells (detectable in green). Histograms show the percentage of BrdU-immunopositive cells among DAPI-positive cells (A), or lipofected cells (B), after cumulative BrdU incorporation; *h* indicates the time of incorporation (in hours); *n* reports the total number of cells counted; and error bars show standard error of the mean. Diagrams show the linear function used to calculate the length of the cell cycle, according to Takahashi et al. [39]; abscissae indicate time (in hours); ordinates show LI of wt (A) or lipofected (B) cells; and *T*c and *T*s estimate the duration in hours of cell cycle and S phase, respectively. *T*c and *T*s of control-lipofected cells (not shown) are virtually the same as those of st. 34 wt cells.
In wt retinas, the proportion of cycling cells, expressed as 10 h LI, is higher at st. 30 (32% ± 0.34% standard error of the mean) than at st. 34 (23% ± 0.1% standard error of the mean). This proportion, at st. 34, is even higher in *cdk2/cyclinA2*–lipofected cells (45% ± 0.1% standard error of the mean) and in *XE2F*-lipofected cells (48% ± 2% standard error of the mean), which both delay the exit from the cell cycle.
In wt retinas, the average cell cycle length, as evaluated by *T*c value, increases from st. 30 (*T*c = 5.1 ± 1.3 h) to st. 34 (*T*c = 8.1 ± 0.6 h). Notably at st. 34, *XE2F* lipofection significantly reduces *T*c (*T*c = 5.5 ± 1.2 h) compared to control cells of the same age (*T*c = 8.1 ± 0.6 h), whereas *T*c is not significantly affected by *cdk2/cyclinA2* lipofection (*T*c = 7.8 ± 0.9 h). The changes in *T*c observed among different types of cells are poorly affected by *T*s, which ranges from 0.9 to 1.4.
(1.7 MB JPG)

Table S1 In Silico Screening for Candidate miRNA Domains Table S1 shows X. laevis (Xla-mir-), Danio rerio (dre-let/mir-), and Homo sapiens (has-mir-) putative miRNA domains present in the 3′ UTR of *Xotx5b, Xvsx1,* and *Xotx2.* Among the 30 X. laevis mature miRNAs so far isolated, 27 (90%) show perfect sequence homology with miRNAs of other species [44]. Because of the low number of annotated *Xenopus* miRNAs and the extreme evolutionary conservation of the mature miRNA sequence, it was reasonable to search also for heterologous domains in the 3′ UTR of the three genes. The MIRanda software [45] was used to screen among the miRNA sequences from the three species annotated in the Sanger miRNA registry (http://microrna.sanger.ac.uk). Only results with energy values lower than −20.00 kcal/mol and score values higher than 100 for at least one of the three UTR are shown. These two thresholds were used in association with gap-open and gap-elongation parameters −8.0 and −2.0, respectively, to ensure high stringency [46]. Sites columns report the number of candidate domains in the corresponding 3′ UTR.
A total number of 42 different miRNAs show in silico high binding affinities for the 3′ UTR of *Xotx5b* (*n* = 20), *Xvsx1* (*n* = 28), and *Xotx2* (*n* = 15), four of them (in bold), sharing sites for all three UTRs. Interestingly, two of these shared miRNAs (dre-mir-34 and dre-mir-432) show multiple domains in Xvsx1 and Xotx2 but not in Xotx5b UTR. miRNA domains are reasonably well dispersed over the three UTR sequences in all three genes under consideration. No obvious nucleotide conservation was found among the three 3′ UTRs. The selected miRNAs are candidates for the translational repression of *Xotx5b, Xvsx1,* and *Xotx2.* However, the presence of the human and D. rerio–selected miRNAs also in *Xenopus,* as well as the expression of all selected miRNAs in developing retina and their hypothetical role in development, remain to be validated.
(20 KB XLS)

### Accession Numbers

The GenBank (http://www.ncbi.nlm.nih.gov/Genbank) accession number for the *Xvsx1* coding sequence is DQ324366. 3′ UTRs discussed in this paper were amplified by RT-PCR based on the following GenBank sequences: *Xotx5b* (BC077545), *Xvsx1* (BC044049), and *Xotx2* (BC077357).

## Abbreviations

CMZ: ciliary marginal zone
GFP: green fluorescent protein
HUA: hydroxyurea/aphydicoline
IRBP: interphotoreceptor retinoid-binding protein
LI: labeling index
miRNA: microRNA
st.: stage
UTR: untranslated region
wt: wild-type

## References

[pbio-0040272-b001] Livesey FJ, Cepko CL (2001). Vertebrate neural cell-fate determination: Lessons from the retina. Nat Rev Neurosci.

[pbio-0040272-b002] McConnell SK, Kaznowski CE (1991). Cell cycle dependence of laminar determination in developing neocortex. Science.

[pbio-0040272-b003] McConnell SK (1995). Constructing the cerebral cortex: Neurogenesis and fate determination. Neuron.

[pbio-0040272-b004] Isshiki T, Pearson B, Holbrook S, Doe CQ (2001). *Drosophila* neuroblasts sequentially express transcription factors which specify the temporal identity of their neuronal progeny. Cell.

[pbio-0040272-b005] Kanai MI, Okabe M, Hiromi Y (2005). Seven-up controls switching of transcription factors that specify temporal identities of *Drosophila* neuroblasts. Dev Cell.

[pbio-0040272-b006] Grosskortenhaus R, Pearson BJ, Marusich A, Doe CQ (2005). Regulation of temporal identity transitions in *Drosophila* neuroblasts. Dev Cell.

[pbio-0040272-b007] Livesey R, Cepko C (2001). Neurobiology. Developing order. Nature.

[pbio-0040272-b008] Cepko CL (1999). The roles of intrinsic and extrinsic cues and bHLH genes in the determination of retinal cell fates. Curr Opin Neurobiol.

[pbio-0040272-b009] Moore KB, Schneider ML, Vetter ML (2002). Posttranslational mechanisms control the timing of bHLH function and regulate retinal cell fate. Neuron.

[pbio-0040272-b010] Hatakeyama J, Kageyama R (2004). Retinal cell fate determination and bHLH factors. Semin Cell Dev Biol.

[pbio-0040272-b011] Dyer MA, Livesey FJ, Cepko CL, Oliver G (2003). Prox1 function controls progenitor cell proliferation and horizontal cell genesis in the mammalian retina. Nat Genet.

[pbio-0040272-b012] Poggi L, Vottari T, Barsacchi G, Wittbrodt J, Vignali R (2004). The homeobox gene *Xbh1* cooperates with proneural genes to specify ganglion cell fate within the *Xenopus* neural retina. Development.

[pbio-0040272-b013] Freund CL, Gregory-Evans CY, Furukawa T, Papaioannou M, Looser J (1997). Cone-rod dystrophy due to mutations in a novel photoreceptor-specific homeobox gene (CRX) essential for maintenance of the photoreceptor. Cell.

[pbio-0040272-b014] Viczian AS, Vignali R, Zuber ME, Barsacchi G, Harris WA (2003). XOtx5b and XOtx2 regulate photoreceptor and bipolar fates in the *Xenopus* retina. Development.

[pbio-0040272-b015] Shen YC, Raymond PA (2004). Zebrafish cone-rod *(crx)* homeobox gene promotes retinogenesis. Dev Biol.

[pbio-0040272-b016] Chow RL, Volgyi B, Szilard RK, Ng D, McKerlie C (2004). Control of late off-center cone bipolar cell differentiation and visual signaling by the homeobox gene *Vsx1*. Proc Natl Acad Sci U S A.

[pbio-0040272-b017] Burmeister M, Novak J, Liang MY, Basu S, Ploder L (1996). Ocular retardation mouse caused by Chx10 homeobox null allele: Impaired retinal progenitor proliferation and bipolar cell differentiation. Nat Genet.

[pbio-0040272-b018] Casarosa S, Amato MA, Andreazzoli M, Gestri G, Barsacchi G (2003). Xrx1 controls proliferation and multipotency of retinal progenitors. Mol Cell Neurosci.

[pbio-0040272-b019] Ohnuma S, Hopper S, Wang KC, Philpott A, Harris WA (2002). Co-ordinating retinal histogenesis: Early cell cycle exit enhances early cell fate determination in the *Xenopus* retina. Development.

[pbio-0040272-b020] Wang JC, Harris WA (2005). The role of combinational coding by homeodomain and bHLH transcription factors in retinal cell fate specification. Dev Biol.

[pbio-0040272-b021] Chang WS, Harris WA (1998). Sequential genesis and determination of cone and rod photoreceptors in *Xenopus*. J Neurobiol.

[pbio-0040272-b022] Nieuwkoop PD, Faber J (1994). The stages of *Xenopus* embryonic development.

[pbio-0040272-b023] Perron M, Kanekar S, Vetter ML, Harris WA (1998). The genetic sequence of retinal development in the ciliary margin of the *Xenopus* eye. Dev Biol.

[pbio-0040272-b024] Mansfield JH, Harfe BD, Nissen R, Obenauer J, Srineel J (2004). MicroRNA-responsive “sensor” transgenes uncover Hox-like and other developmentally regulated patterns of vertebrate microRNA expression. Nat Genet.

[pbio-0040272-b025] Xie X, Lu J, Kulbokas EJ, Golub TR, Mootha V (2005). Systematic discovery of regulatory motifs in human promoters and 3′ UTRs by comparison of several mammals. Nature.

[pbio-0040272-b026] Yekta S, Shih IH, Bartel DP (2004). MicroRNA-directed cleavage of HOXB8 mRNA. Science.

[pbio-0040272-b027] Boy S, Souopgui J, Amato MA, Wegnez M, Pieler T (2004). XSEB4R, a novel RNA-binding protein involved in retinal cell differentiation downstream of bHLH proneural genes. Development.

[pbio-0040272-b028] Mazumder B, Seshadri V, Fox PL (2003). Translational control by the 3′-UTR: The ends specify the means. Trends Biochem Sci.

[pbio-0040272-b029] Wilkie GS, Dickson KS, Gray NK (2003). Regulation of mRNA translation by 5′- and 3′-UTR-binding factors. Trends Biochem Sci.

[pbio-0040272-b030] Harris WA, Hartenstein V (1991). Neuronal determination without cell division in *Xenopus* embryos. Neuron.

[pbio-0040272-b031] Ohnuma S, Philpott A, Wang K, Holt CE, Harris WA (1999). p27Xic1, a Cdk inhibitor, promotes the determination of glial cells in *Xenopus* retina. Cell.

[pbio-0040272-b032] Gerber WV, Yatskievych TA, Antin PB, Correia KM, Conlon RA (1999). The RNA-binding protein gene, *hermes,* is expressed at high levels in the developing heart. Mech Dev.

[pbio-0040272-b033] Gonzalez-Fernandez F, Kittredge KL, Rayborn ME, Hollyfield JG, Landers RA (1993). Interphotoreceptor retinoid-binding protein (IRBP), a major 124 kDa glycoprotein in the interphotoreceptor matrix of *Xenopus laevis.* Characterization, molecular cloning and biosynthesis. J Cell Sci.

[pbio-0040272-b034] Candal E, Thermes V, Joly JS, Bourrat F (2004). Medaka as a model system for the characterisation of cell cycle regulators: A functional analysis of Ol-Gadd45gamma during early embryogenesis. Mech Dev.

[pbio-0040272-b035] de la Calle-Mustienes E, Glavic A, Modolell J, Gomez-Skarmeta JL (2002). Xiro homeoproteins coordinate cell cycle exit and primary neuron formation by upregulating neuronal-fate repressors and downregulating the cell-cycle inhibitor XGadd45-gamma. Mech Dev.

[pbio-0040272-b036] Logan MA, Steele MR, Van Raay TJ, Vetter ML (2005). Identification of shared transcriptional targets for the proneural bHLH factors Xath5 and XNeuroD. Dev Biol.

[pbio-0040272-b037] Caviness VS, Takahashi T, Nowakowski RS (1995). Numbers, time and neocortical neuronogenesis: A general developmental and evolutionary model. Trends Neurosci.

[pbio-0040272-b038] Alexiades MR, Cepko C (1996). Quantitative analysis of proliferation and cell cycle length during development of the rat retina. Dev Dyn.

[pbio-0040272-b039] Takahashi T, Nowakowski RS, Caviness VS (1995). The cell cycle of the pseudostratified ventricular epithelium of the embryonic murine cerebral wall. J Neurosci.

[pbio-0040272-b040] Tanaka T, Ono T, Kitamura N, Kato JY (2003). Dominant negative E2F inhibits progression of the cell cycle after the midblastula transition in *Xenopus*. Cell Struct Funct.

[pbio-0040272-b041] Holt CE, Garlick N, Cornel E (1990). Lipofection of cDNAs in the embryonic vertebrate central nervous system. Neuron.

[pbio-0040272-b042] Suzuki A, Hemmati-Brivanlou A (2000). *Xenopus* embryonic E2F is required for the formation of ventral and posterior cell fates during early embryogenesis. Mol Cell.

[pbio-0040272-b043] Gross SP, Tuma MC, Deacon SW, Serpinskaya AS, Reilein AR (2002). Interactions and regulation of molecular motors in *Xenopus* melanophores. J Cell Biol.

[pbio-0040272-b044] Watanabe T, Takeda A, Mise K, Okuno T, Suzuki T (2005). Stage-specific expression of microRNAs during *Xenopus* development. FEBS Lett.

[pbio-0040272-b045] Enright AJ, John B, Gaul U, Tuschl T, Sander C (2003). MicroRNA targets in *Drosophila.*. Genome Biol.

[pbio-0040272-b046] John B, Enright AJ, Aravin A, Tuschl T, Sander C (2004). Human microRNA targets. PLoS Biol.

[pbio-0040272-b047] Huang S, Moody SA (1998). Dual expression of GABA or serotonin and dopamine in *Xenopus* amacrine cells is transient and may be regulated by laminar cues. Vis Neurosci.

[pbio-0040272-b048] Vigh J, Banvolgyi T, Wilhelm M (2000). Amacrine cells of the anuran retina: Morphology, chemical neuroanatomy, and physiology. Microsc Res Tech.

